# Establishment of a prognostic prediction system based on tumor microenvironment of pancreatic cancer

**DOI:** 10.1097/MD.0000000000032364

**Published:** 2022-12-23

**Authors:** Yan Feng, Pengcheng Li, Fang Yang, Ke Xu

**Affiliations:** a Department of Hepatology, the Affiliated Hospital of Panzhihua University, Sichuan, China; b Clinical Medical College, Chengdu Medical College, Sichuan, China; c Department of Oncology, The First Affiliated Hospital of Chengdu Medical College, Sichuan, China; d Key Clinical Specialty of Sichuan Province, Sichuan, China.

**Keywords:** overall survival, pancreatic cancer, prognostic system, risk score, tumor microenvironment

## Abstract

Pancreatic cancer (PC) is an inflammatory tumor. Tumor microenvironment (TME) plays an important role in the development of PC. This study aims to explore hub genes of TME and establish a prognostic prediction system for PC.

**Methods:**

High throughput RNA-sequencing and clinical data of PC were downloaded from The Cancer Genome Atlas and International Cancer Genome Consortium database, respectively. PC patients were divided into high- and low-score group by using stromal, immune scores system based on ESTIMATE. Differentially expressed genes between high- and low-score patients were screened and survival-related differentially expressed genes were identified as candidate genes by univariate Cox regression analysis. Final variables for establishment of the prognostic prediction system were determined by LASSO analysis and multivariate Cox regression analysis. The predictive power of the prognostic system was evaluated by internal and external validation.

**Results:**

A total of 210 candidate genes were identified by stromal, immune scores system, and survival analyses. Finally, the prognostic risk score system was constructed by the following genes: FAM57B, HTRA3, CXCL10, GABRP, SPRR1B, FAM83A, and LY6D. In process of internal validation, Harrell concordance index (C-index) of this prognostic risk score system was 0.73, and the area under the receiver operating characteristic curve value of 1-year, 2-year, and 3-year overall survival period was 0.67, 0.76 and 0.86, respectively. In the external validation set, the survival prediction C-index was 0.71, and the area under the curve was 0.81, 0.72, and 0.78 at 1-year, 2-year, and 3-year, respectively.

**Conclusion:**

This prognostic risk score system based on TME demonstrated a good predictive capacity to the prognosis of PC. It may provide information for the treatment strategy and follow-up for patients with PC.

## 1. Introduction

Pancreatic cancer (PC) is the seventh leading cause of death and may even rise to third in the future.^[1]^ In contrast to the overall declining trends for other malignancies, the stable mortality rate of PC is considered to be caused by the slow progress of diagnosis and treatment in recent years.^[2]^ Currently, surgery is still the only radical treatment for PC, and TNM stage is the most widely used prognosis indicator for PC patients. Many factors, such as positive margin, adjuvant chemotherapy, postoperative complications and back pain were also found to be significantly related to the prognosis of PC.^[3–5]^ It is crucial to make an accurately prognosis prediction for making individual treatment plan to PC patients. Therefore, it is necessary to use different principles to explore rational and optimized prognosis models.

Tumor microenvironment (TME) is composed of tumor cells, inflammatory cells, endothelial cells, hematopoietic cells, fibroblasts, extracellular matrix and various bioactive secretions.^[6]^ In the field of PC, it has been found that the surrounding microenvironment of PC plays an important role in tumor proliferation, immune escape, drug resistance and metastasis, thus affecting the prognosis of the patients.^[7–14]^ Different tumor samples were given immune score and stromal score based on the expression of relevant genes involved in TME to help us explore potential information about TME by ESTIMATE.^[15]^ In this study, we used ESTIMATE to mine the genes related to TME, and construct a prognostic risk score system based on these genes for PC.

## 2. Methods

### 2.1. Data acquisition

RNA sequencing and clinical data (age, gender, differentiation, T staging, N staging, and survival data) of PC patients were downloaded from The Cancer Genome Atlas (TCGA). A total of 170 patients were included in this study after exclusion of the patients with overall survival (OS) < 30 days. Subsequently, stromal and immune scores of these 170 PC patients were obtained from ESTIMATE (https://bioinformatics.mdanderson.org/estimate/).^[15]^ And then, RNA sequencing and survival data of PC patients were also downloaded from International Cancer Genome Consortium (ICGC) (https://dcc.icgc.org/). A total of 63 patients were constituted the external validation data set for the study after exclusion of the patients with OS < 30 days.

### 2.2. Identification of candidate genes

Kaplan–Meier method was used to assess OS of patients with different stromal and immune scores. X-Tile software was used to determine the best thresholds for grouping respectively.^[16]^ The limma package was used to analyze the differentially expressed genes (DEGs) between the groups with high and low stromal scores, and high and low immune scores.^[17]^ Fold changes (FC) |log2FC| ≥ 1 and adjusted *P* < .05 was considered had statistically significant difference. Co-DEGs were obtained by taking intersection of these two DEGs sets. Finally, survival related DEGs were screened by univariate Cox analysis and identified as candidate genes.

### 2.3. Gene Ontology (GO) term enrichment analysis, Kyoto Encyclopedia of Genes and Genomes (KEGG) pathway analysis

DAVID online tool (https://david.ncifcrf.gov/), ClusterProfiler package were used to carry out GO and KEGG enrichment analysis by inputting candidate genes.^[18,19]^ GO analysis includes three parts: cellular component (CC), molecular function (MF) and biological process (BP). Visualize the analysis results through the GOplot package.^[20]^ The two terms with adjusted *P* < .05 were considered to be statistically significant.

### 2.4. Establishment and validation of prognostic risk score system

One hundred seventy PC patients from TCGA were divided into two groups in a ratio of 3:2. The process of grouping was in accordance with the principle of randomization. One group with 102 patients was set as the training set, and another group with 68 patients was set as the internal validation set. LASSO analysis was performed by using glmnet package to narrow down candidate genes.^[21]^ Stepwise method was adopted in multivariate Cox regression analysis to determine the final genes for establishment of the prognostic risk score system. The risk score calculation formula was as follows:

Risk score =β1 * Expβ1 +β2 * Expβ2 + β3 * Expβ3 + βi * Expβi

The prognostic risk score system was validated in training set, internal validation set and external validation set. The performance of the risk score system was investigated by calculation of the Harrell’s concordance index (C-index).^[22]^ TimeROC package was used for construction of the time dependent receiver operating characteristic (ROC) and calculation of the area under the ROC curve (AUC) at 1-year, 2-year and 3-year OS.^[23]^ Univariate and multivariate Cox regression survival analysis were performed by adding the factor of risk score in the training set.

### 2.5. Relationship between risk score system and clinical characteristics, immune cell infiltration and immune checkpoint coding genes (ICGs)

Wilcoxon test was used to compare the difference of risk score between different characteristics of clinical parameters. 170 PC patients from TCGA were divided into high- and low-risk group based on the median prognostic score. The difference of 22 immune cell infiltration between the two groups was analyzed by CIBERSORT algorithm.^[24]^ Furthermore, the expression of ICGs between high- and low-risk group were also investigated.

### 2.6. Statistical analysis

R software v3.6.3 (R Project for Statistical Computing, Vinenna, Austria) (www.r-project.org) was used for statistical analyses in this study. The numerical variables were described as means ± SD. Kaplan–Meier method was used in survival analysis, and log-rank test was used to compare the differences between groups. Wilcoxon test was used for nonparametric comparisons. All statistical tests were two-tailed, *P* < .05 were considered to be significantly different.

## 3. Results

### 3.1. Basic information of the PC patients

One hundred and seventy PC patients with completed information were extracted from TCGA. The age was 64.45 ± 10.83 years old, the proportion of males was 54.1%, median OS was 15.35 months. After random grouping according to 3:2, the age of the training set was 63.08 ± 10.23 years old, the proportion of males was 54.9%, and median OS was 15.27 months. The age of the internal validation set was 66.51 ± 11.44 years, the proportion of males was 52.9% males, and median OS was 15.68 months. 63 PC patients from ICGC had a median OS of 14.10 months. The detail of each clinical parameter was shown in Table 1.

**Table 1:** The clinical characteristics of PC patients.

Clinical characteristic	TCGA training set		TCGA internal validation set		ICGC external validation set	
	Total (n = 102)	Percent (%)	Total (n = 68)	Percent (%)	Total (n = 63)	Percent (%)
Age (mean ± SD)	63.08 ± 10.23		66.51 ± 11.44		65.89 ± 11.24	
Gender						
Male	56	54.9	36	52.9	31	49.2
Female	46	45.1	32	47.1	32	50.8
Grade						
G1 + G2	74	72.5	45	66.2	40	63.5
G3 + G4	28	27.5	21	30.9	22	34.9
T stage						
T1 + T2	13	12.7	14	20.6	4	6.3
T3 + T4	89	87.3	52	76.5	57	90.5
N status						
N0	24	23.5	23	33.8	16	25.4
N+	73	71.6	41	60.3	45	71.4
NA	5	4.9	4	5.9	2	3.2
TNM stage						
I + II	95	93.1	65	95.6	0	0
III + IV	7	6.9	1	1.5	0	0
NA	0	0	2	2.9	63	100
Survival status						
Alive	48	47.1	32	47.1	26	41.3
	54	52.9	36	52.9	37	58.7

PC = pancreatic cancer.

### 3.2. Grouping and survival analysis based on immune and stromal scores

**Figure 1.**
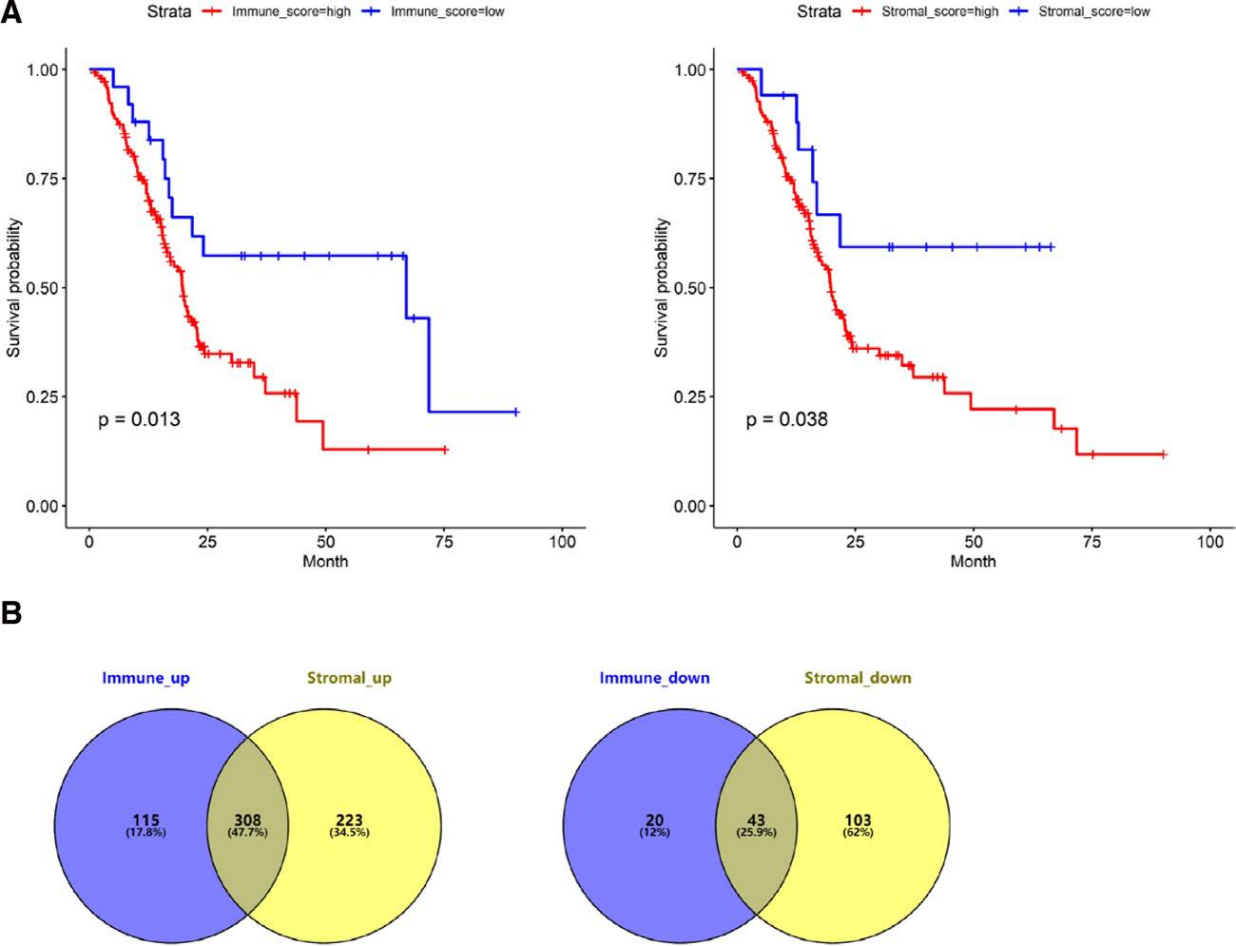
Survival analysis based on immune and stromal scores and identification of co-DEGs. (A) Kaplan–Meier survival curves of high and low immune score group, and high and low stromal scores group. (B) The Venn diagram of significantly up-regulated and down-regulated genes. DEGs = differentially expressed genes.

-180.59 was calculated as the best cutoff value for grouping based on immune scores, and there was a significant difference in OS between high and low immune score group (*P* = .013). In the same way, -542.33 was set as the best cutoff value for grouping based on stromal scores, and there was also a significant difference in OS between the high and low stromal scores group (*P* = .038). As shown in Figure 1A.

### 3.3. Identification of candidate genes

By analyzing the DEGs between high and low immune score group, a total of 486 DEGs were identified, including 423 up-regulated genes and 63 down-regulated genes. 531 up-regulated genes and 146 down-regulated genes were screened when high and low stromal scores group was compared. A total of 351 DEGs were obtained after taking intersection of the two DEGs sets, including 308 up-regulated genes and 43 down-regulated genes. Finally, 210 candidate genes were identified by univariate Cox regression analysis. As shown in Figure 1B.

### 3.4. GO enrichment analysis and KEGG pathway enrichment analysis

**Figure 2.**
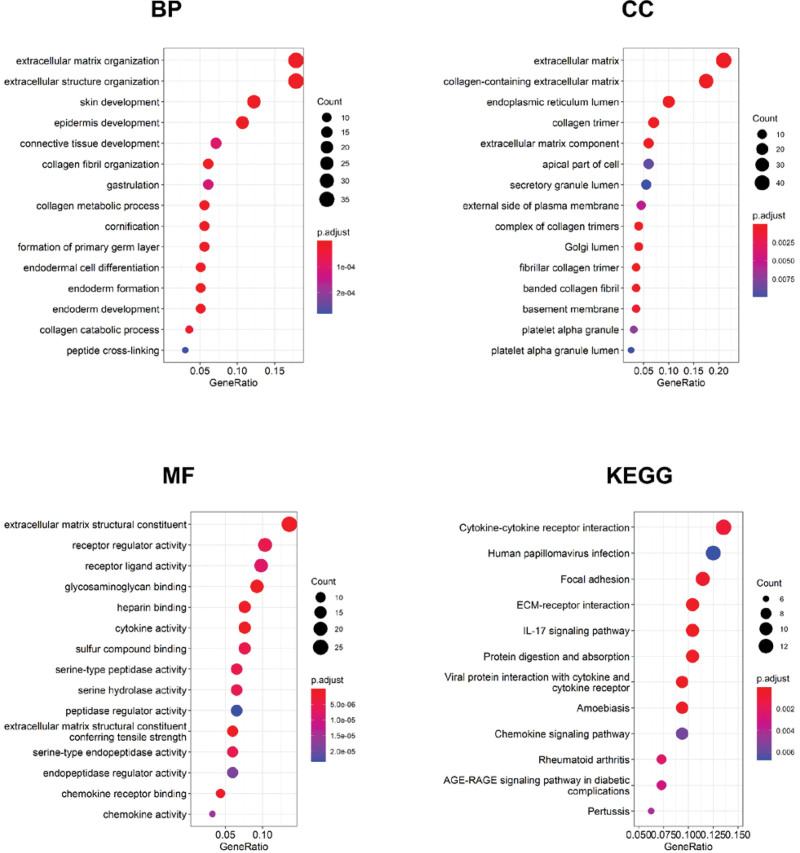
GO and KEGG enrichment analysis of the 210 candidate genes. GO = Gene Ontology, KEGG = Kyoto Encyclopedia of Genes and Genomes.

When GO enrichment analysis was carried out on 210 candidate genes of PC, extracellular matrix organization, extracellular matrix and extracellular matrix structural constituent was the most significant functional enrichment of BP, CC and MF, respectively. Cytokine-cytokine receptor interaction was the most relevant pathway by KEGG enrichment analysis. See Figure 2 and Table 2.

**Table 2:** GO and KEGG enrichment analysis of candidate TME related genes in PC ranked by p value. (TOP 5).

Category	GO or KEGG ID	GO or KEGG term	p.adjust	Count
BP	GO:0030198	Extracellular matrix organization	5.60E-21	35
BP	GO:0043062	Extracellular structure organization	3.92E-19	35
BP	GO:0030199	Collagen fibril organization	3.72E-11	12
BP	GO:0043588	Skin development	1.60E-08	24
BP	GO:0035987	Endodermal cell differentiation	2.27E-08	10
CC	GO:0031012	Extracellular matrix	1.49E-24	42
CC	GO:0062023	Collagen-containing extracellular matrix	4.45E-20	35
CC	GO:0044420	Extracellular matrix component	4.03E-12	12
CC	GO:0005581	Collagen trimer	1.87E-11	14
CC	GO:0005583	Fibrillar collagen trimer	1.52E-10	7
MF	GO:0005201	Extracellular matrix structural constituent	9.34E-20	25
MF	GO:0030020	Extracellular matrix structural constituent conferring tensile strength	5.99E-11	11
MF	GO:0005539	Glycosaminoglycan binding	5.27E-09	17
MF	GO:0008201	Heparin binding	5.47E-08	14
MF	GO:0005125	Cytokine activity	7.40E-08	14
KEGG	hsa04512	ECM-receptor interaction	8.21E-06	10
KEGG	hsa04657	IL-17 signaling pathway	8.21E-06	10
KEGG	hsa04974	Protein digestion and absorption	8.21E-06	10
KEGG	hsa04061	Viral protein interaction with cytokine and cytokine receptor	9.50E-05	9
KEGG	hsa05146	Amoebiasis	9.50E-05	9

BP = biological process, CC = cellular component, GO = Gene Ontology, KEGG = Kyoto Encyclopedia of Genes and Genomes, MF = molecular function, PC = pancreatic cancer, TME = tumor microenvironment.

### 3.5. Establishment of the prognostic risk score system

**Figure 3.**
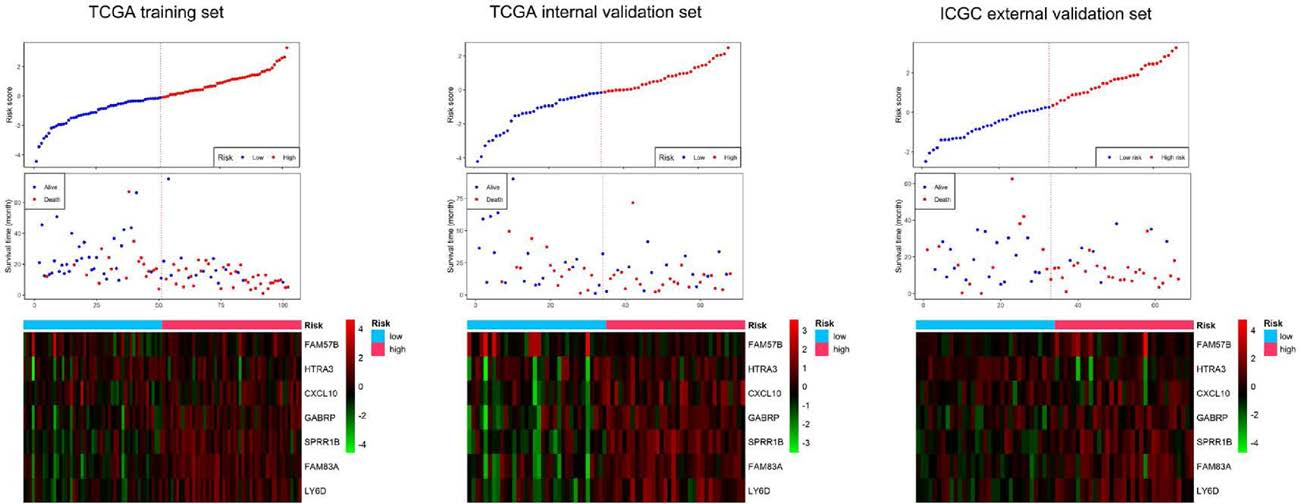
Risk score distribution, survival status, and expression patterns of 7 included genes in risk score system for high- and low-risk patients from training set, internal validation set and external validation set.

A total of 12 genes were screened from the 210 candidate genes by using LASSO analysis, and final 7 variables were determined for construction the prognostic risk score system by multivariate Cox regression analysis, as shown in Figure 3. The calculation formula was as follows:

Risk score = (0.331 * Exp FAM57B) + (-0.375 * Exp HTRA3) + (0.320 * Exp CXCL10) + (0.205 * Exp GABRP) + (0.139 * Exp SPRR1B) + (0.217 * Exp FAM83A) + (0.092 * Exp LY6D)

### 3.6. Validation of the prognostic risk score system

**Figure 4.**
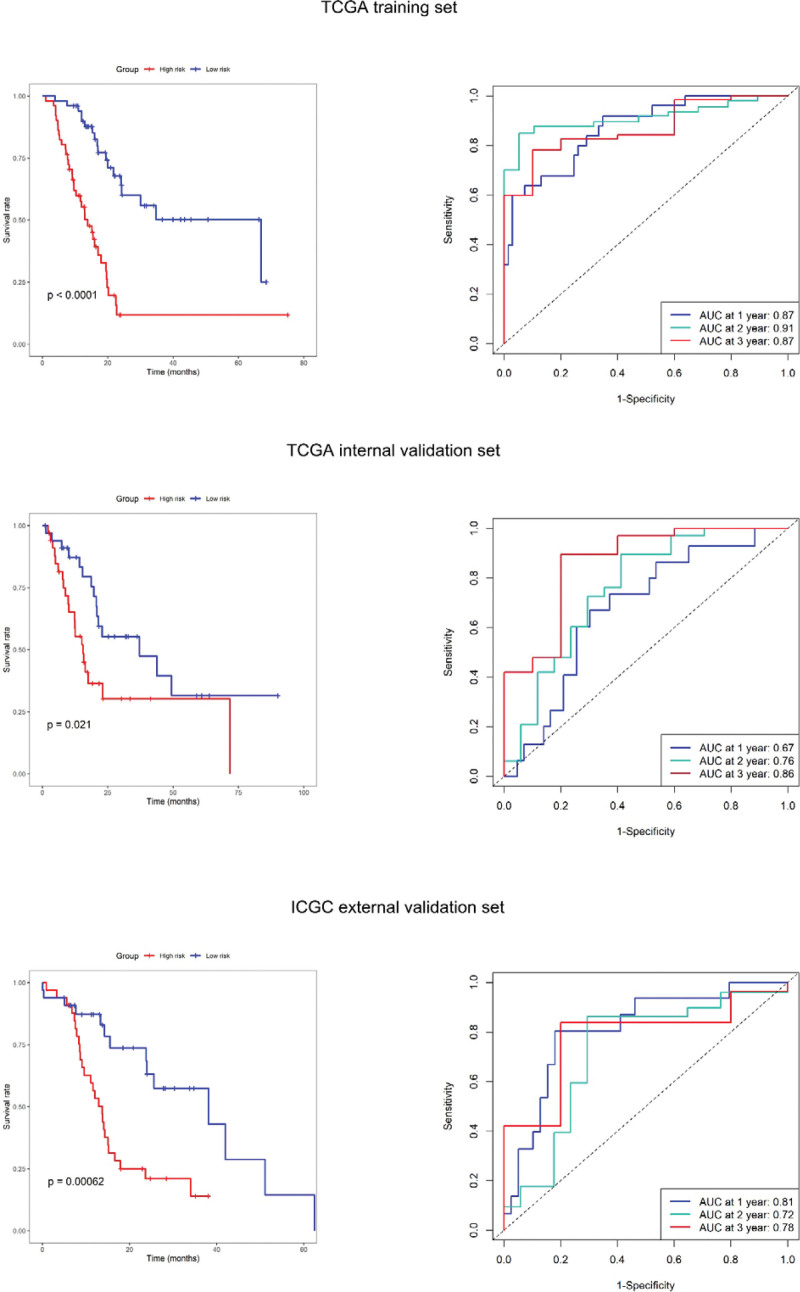
Kaplan–Meier survival curves of high- and low-risk patients and the time dependent ROC at 1-year, 2-year, and 3-year OS from verification set, internal validation set and external validation set. ROC = receiver operating characteristic.

**Figure 5.**
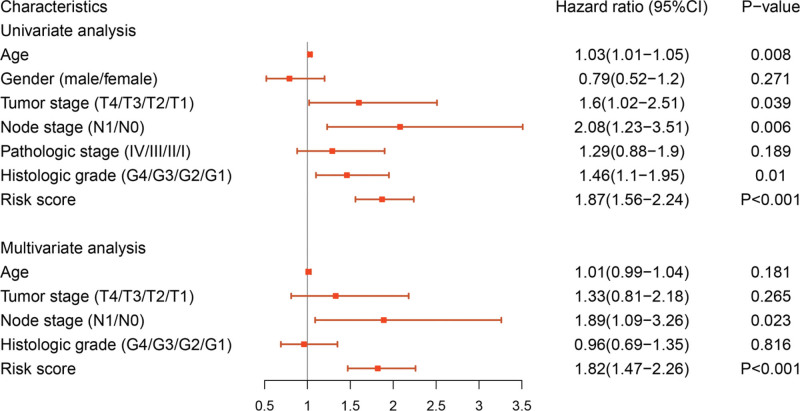
Univariate and multivariate Cox regression analysis of OS.

Patients were divided into high- and low-risk group by setting the median as the cutoff value in each data set. It was found that the high- and low-risk group all had significant survival differences in training set, internal validation and external validation (*P* = .000, *P* = .021, *P* = .001). In the training set, Harrell’s C-index of this prognostic risk score system was 0.78, and the AUC was 0.87, 0.91, 0.87 at 1-year, 2-year and 3-year, respectively. In process of internal validation, the survival prediction C-index was 0.73, and AUC value of 1-year, 2-year and 3-year OS period was 0.67, 0.76 and 0.86, respectively. In the external validation set, the survival prediction C-index was 0.71, and the AUC was 0.81, 0.72, 0.78 at 1-year, 2-year and 3-year, respectively. As shown in Figure 4. Univariate and multivariate regression analyses were carried out by adding the factor of risk score in the training set. Only risk score was independent factors for OS (*P* < .001). As shown in Figure 5.

### 3.7. Relationship between risk score system and clinical characteristics, immune cell infiltration and ICGs

**Figure 6.**
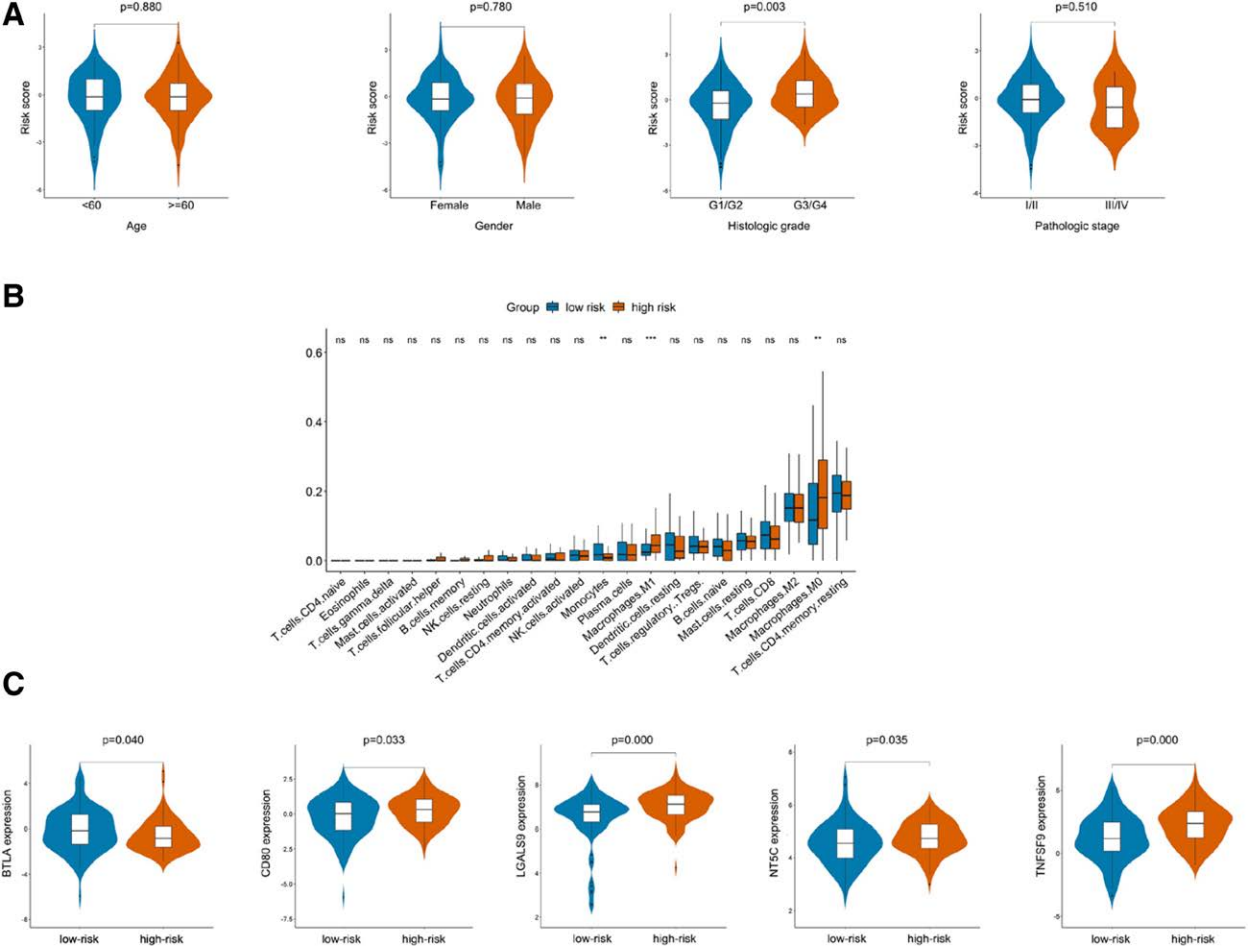
Relationship between risk score system and clinical characteristics (A), immune cell infiltration (B) and the expression of ICGs (C). ICGs = immune checkpoint coding genes.

PC patients with the differentiation of G3/G4 had a higher prognostic risk score than patients with G1/G2 (*P* = .003), and no significant difference was found among patients with different age, gender and TNM staging. The proportions of M0 and M1 macrophage were higher in high risk score patients, while the proportion of monocytes was less than the patients with low risk score (*P* = .008, *P* = .001, *P* = .008). The prognostic risk score was significantly correlated with the expression of the following ICGs: BTLA, CD80, LGALS9, NT5C, TNFSF9 (*P* = .040, *P* = .033, *P* = .000, *P* = .035, *P* = .000). As shown in Figure 6A‐C.

## 4. Discussion

The prognosis of PC was extremely poor. The overall 5-year survival rate was only 9%, and merely 37% in stage I or IIA postoperative PC patients.^[2]^ There were many prognostic factors for PC patients, and it was difficult to predict the prognosis accurately by relying on solo prognostic indicator. Some prognostic prediction systems were constructed with clinical parameters to improve the predictive capability, classical ones such as Botsis score, Heidelberg score, MSKCC score, etc.^[3,25,26]^ However, their performances were still not entirely unsatisfactory. More accurate indicators and prediction systems should be used as the reference for individualized clinical decision-making in the future. As the mechanism of TME was gradually revealed, it was a reasonable and feasible way to explain clinical characteristics and improve clinical practice guide by using the information of TME. ESTIMATE score system was developed in 2013. The principle was to use the gene expression data of immune and stromal cells to infer the contents of them in tumor samples.^[15]^ Currently, RNA sequencing data of 25 cancer species from TCGA had been analyzed by ESTIMATE. In this study, a total of 210 candidate genes related to TME and prognosis were identified by using ESTIMATE and survival analysis. These genes were significantly enriched in the construction of extracellular matrix and Cytokine-cytokine receptor interaction by GO and KEGG enrichment analysis. Finally, seven genes were screened for the construction of the prognostic risk score system which was rare reported. Now, these genes would be briefly described below. The mechanism of FAM57B and LY6D in malignant tumors has not been reported. HTRA3, which expresses a pro-apoptotic protease, promotes drug-induced cytotoxic effects and was thought to have the function of anti-tumor.^[27]^ It showed inhibition of tumor proliferation and metastasis in lung cancer cells and endometrial cancer cells, and high expression of HTRA3 was associated with longer disease-free survival in non-small cell lung cancer patients.^[28–30]^ SPRR1B is overexpressed in oral tumor stem cells and has been found to induce tumor proliferation by activating MAPK signaling, but its specific functional mechanism in PC has not been investigated.^[31]^ CXCL10, also known as IP-10, high expression in the TME of PC could influence lymphocytes recruitment and was correlate with poor survival.^[32]^ GABRP, significantly higher expression in PC tissue than normal tissues, could promote tumor growth and metastasis. Higher expression of GABRP was also associated with poor prognosis of PC. In mechanism, GABRP interacts with KCNN4 to induce calcium influx, activate the nuclear factor κB signaling, and ultimately promote macrophage infiltration by inducing the expression of CXCL5 and CCL20.^[33]^ Overexpression of FAM83A activated TGF-β signaling pathway and the Wnt/β-catenin signaling. In vitro and vivo, FAM83A expression was associated with the characteristics of PC tumor stem cell and the generation of chemotherapy resistance.^[34]^ The role of these novel genes in tumorigenesis and tumor development should be further investigated.

The predictive capabilities of the prognostic risk score systems were different by construction using different principles and angles. In the past decade, with the rapid development of bioinformatics, tumor molecular biology and tumor immunology, a large number of molecular markers have been discovered and were considered to have potential diagnostic and therapeutic value. The number of the prognostic models constructed by tumor molecular markers were gradually increasing, and most of them showed acceptable performance.^[35–39]^ However, there was still no perfect prognostic model due to the heterogeneous tumors and unknown pathogenesis. Current research on the pathogenesis of malignant tumors, a large number of TME related molecular markers have been revealed had the relationship with tumor proliferation, immune escape, drug resistance, metastasis and so on. They also play as mediators for tumor - environment interaction. Many researchers have transferred their focuses from tumor cells to the surrounding environment of tumor cells. PC, as an inflammatory tumor, has changes in the TME during its development of inflammatory - cold process.^[14,40,41]^ In clinical research on TME and PC, phase Ib study of PEGylated human recombinant hyaluronidase (PEGPH20) in combination with gemcitabine in advanced PC patients has shown promising efficacy.^[42]^ Phase I study of CCR2 inhibition in combination with FOLFIRINOX in borderline resectable and locally advanced PC patients also has yielded a good clinical outcome.^[43]^ It is very important to study TME related prognostic genes of PC, which can further reveal the tumor development and progression. Establishment of a prognostic system based on TME related prognostic genes can more accurately and conveniently evaluate the prognosis of PC patients. Pu et al analyzed the TME related prognostic genes through the similar way, and identified 53 genes related to immune score and 17 genes related to stromal score, which were related to recurrence free survival. Unfortunately, prognostic model had not further constructed.^[44]^ To our knowledge, this prognostic prediction system was the first model constructed by ESTIMATE for PC. According to this prognostic risk score system, Harrell’s C-index was 0.73, and AUC value of 1-year, 2-year and 3-year OS period was 0.67, 0.76 and 0.86 in the internal validation set. Harrell’s C-index was 0.71, and AUC value of 1-year, 2-year and 3-year OS period was 0.81, 0.72 and 0.78 in the external validation set. By reviewing other reported prognostic prediction systems of PC, this prognostic system contains fewer variables and has a good predictive capacity.^[35–38]^ Furthermore, it was found this prognostic risk score system was significantly associated with the expression of some ICGs, suggesting that it may have predictive implications for immunotherapy response and efficacy.

Since the data were based on TCGA and ICGC, the limited sample size and the quality of data set were the biggest bottleneck of this study. In addition, the functions and mechanisms of the novel genes should be further explored by basic experiments. The effectiveness of this prognostic prediction system needs to be confirmed by multi-center and prospective research.

## 5. Conclusion

This prognostic risk score system based on TME demonstrated a good predictive capacity to the prognosis of PC. It may provide information for the treatment strategy and follow-up for patients with PC.

## Author contributions

**Conceptualization:** Ke Xu.

**Data curation:** Pengcheng Li.

**Investigation:** Fang Yang.

**Methodology:** Ke Xu.

**Software:** Pengcheng Li.

**Visualization:** Fang Yang.

**Writing – original draft:** Yan Feng.
